# Optimized Photodynamic Therapy with Multifunctional Cobalt Magnetic Nanoparticles

**DOI:** 10.3390/nano7060144

**Published:** 2017-06-10

**Authors:** Kyong-Hoon Choi, Ki Chang Nam, Un-Ho Kim, Guangsup Cho, Jin-Seung Jung, Bong Joo Park

**Affiliations:** 1 Department of Electrical & Biological Physics, Kwangwoon University, Nowon-gu, Seoul 139-701, Korea; solidchem@hanmail.net (K.-H.C.); gscho@kw.ac.kr (G.C.); 2 Department of Medical Engineering, Dongguk University College of Medicine, Gyeonggi-do 10326, Korea; kichang.nam@gmail.com; 3 Department of Chemistry, Gangneung-Wonju National University, Gangneung 210-702, Korea; rladydanr06@naver.com

**Keywords:** photodynamic therapy, optimized nano-carrier, multifunctional magnetic nanoparticle, fluence, anticancer activity, prostate cancer cell

## Abstract

Photodynamic therapy (PDT) has been adopted as a minimally invasive approach for the localized treatment of superficial tumors, representing an improvement in the care of cancer patients. To improve the efficacy of PDT, it is important to first select an optimized nanocarrier and determine the influence of light parameters on the photosensitizing agent. In particular, much more knowledge concerning the importance of fluence and exposure time is required to gain a better understanding of the photodynamic efficacy. In the present study, we synthesized novel folic acid-(FA) and hematoporphyrin (HP)-conjugated multifunctional magnetic nanoparticles (CoFe_2_O_4_-HPs-FAs), which were characterized as effective anticancer reagents for PDT, and evaluated the influence of incubation time and light exposure time on the photodynamic anticancer activities of CoFe_2_O_4_-HPs-FAs in prostate cancer cells (PC-3 cells). The results indicated that the same fluence at different exposure times resulted in changes in the anticancer activities on PC-3 cells as well as in reactive oxygen species formation. In addition, an increase of the fluence showed an improvement for cell photo-inactivation. Therefore, we have established optimized conditions for new multifunctional magnetic nanoparticles with direct application for improving PDT for cancer patients.

## 1. Introduction

Over the last few decades, photosensitizer (PS)-mediated photodynamic therapy (PDT) has been introduced as a possible alternative non-invasive localized therapeutic modality for treating cancer as well as cardiovascular, ophthalmic, dermatological, and dental diseases [1,2,3,4,5,6,7]. PDT is a two-step procedure that involves the administration of a photosensitizing agent [8], followed by activation of the drug with non-thermal light of a specific wavelength [9]. In particular, this photodynamic process rapidly generates reactive oxygen species (ROS) including peroxides, hydroxyl radicals, superoxide ions, and singlet oxygen, with the latter implicated as the major causative agent of cellular damage in the photodynamic process [10]. However, the results of recent clinical and preclinical studies of PDT indicate that this process still suffers from disadvantages such as the wavelength-dependent tissue penetration depth of the light; inefficient delivery of PS to the target area; loss of PDT efficacy owing to PS aggregation, degradation, or reduction; and toxicity of the PS [11,12,13].

Several approaches have been proposed to enhance the efficacy of PDT. In some cases, PDT efficacy was found to be significantly improved when nanoparticles were applied as PS carriers, suggesting that the use of nanoparticles can help to overcome the aforementioned limitations [14,15,16]. Among the various nanoparticles available, such as liposomal vesicles, quantum dots, nanotubes, and gold nanoparticles, the latter have attracted substantial attention because of their chemical inertness, excellent optical properties, and minimal biological toxicity [17,18]. Recently, new synergistic treatment modalities that combine PDT with hyperthermia by using Au nanocomposites have shown the potential to overcome the current limitations of PDT and enhance anticancer efficacy [19,20,21]. However, the Au nanocomposites must overcome many disadvantages, including higher cost, low conjugation efficiency on the surface of particles, and lack of bio-imaging capability. To improve PDT efficacy, it is also important to understand the photophysical and photochemical properties of as-prepared photosensitizing agents. In particular, the illumination parameters might play an important role in determining PDT efficacy.

Herein, we report the development of new multifunctional magnetic nanoparticles conjugated with hematoporphyrin (HP) and folic acid (FA) (CoFe_2_O_4_-HPs-FAs) for use as potential PDT agents, which were tested by targeting prostate cancer PC-3 cells with FA. The biocompatibility and photodynamic anticancer activity of the CoFe_2_O_4_-HPs-FAs were evaluated in vitro. In addition, we evaluated the effect of variations in the fluence and exposure time on the outcome of the photodynamic anticancer activity of CoFe_2_O_4_-HPs-FAs in PC-3 cells to corroborate the importance of optimizing the irradiation parameters.

## 2. Results and Discussion

### 2.1. Characteristics of Multifunctional CoFe_2_O_4_-HPs-Fas

**Scheme 1:**
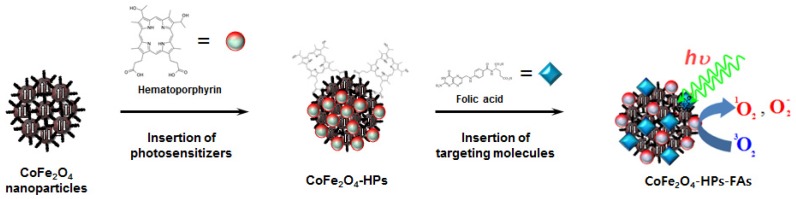
Fabrication procedure for the multifunctional magnetic nanoparticles.

As illustrated in Scheme 1, novel multifunctional magnetic nanoparticles (CoFe_2_O_4_-HPs-FAs) were prepared by simple surface modification of magnetic nanoparticles with a photosensitizer, HP, and a targeting molecule, FA. First, two carboxyl terminal groups of HP are chemically bonded to metal cations on the surface of the CoFe_2_O_4_ nanoparticles via esterification reaction. Similarly, the FA molecules were introduced to the surface of the CoFe_2_O_4_ nanoparticles to improve the targeting ability.

**Figure 1:**
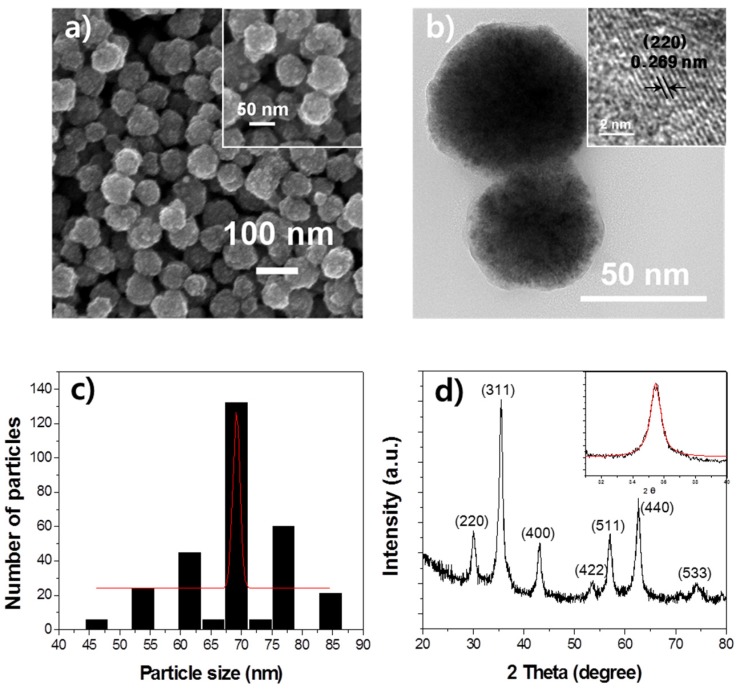
Morphology and crystal structure of the CoFe_2_O_4_ nanoparticle. (**a**) Field-emission scanning electron microscopy image and (**b**) transmission electron microscopy micrographs of the CoFe_2_O_4_ nanoparticle; (**c**) Histogram for the particle size distribution of the CoFe_2_O_4_ nanoparticles; (**d**) X-ray diffraction pattern of the CoFe_2_O_4_ nanoparticles.

The morphology and particle size of the as-prepared CoFe_2_O_4_ nanoparticles were characterized by transmission electron microscopy (TEM; JEOL, JEM-2100F) and scanning electron microscopy (SEM; Hitachi, SU-70), as shown in Figure 1a,b, respectively. The SEM and TEM images showed that these nanoparticles composed of irregular nanograins are spherical and have a diameter of approximately 70 nm with a rough surface. In addition, the sizes of these nanoparticles were quite uniform. The high-resolution TEM image on the edge of a nanoparticle indicated that the distance between two neighboring planes was 0.269 nm at (220), which is in good agreement with the (220) plane of the spinel CoFe_2_O_4_, as shown in the inset of Figure 1b. Figure 1c shows a histogram of the distribution of the nanoparticles size with a Gaussian fit curve (solid line); the particle size ranged from 45 to 85 nm, and the average particle size (*D*_SEM_), defined as the size at the peak of the Gaussian-fitting curve, was 69.2 nm. These results indicated that our CoFe_2_O_4_ nanoparticles were well dispersed and had a narrow size distribution.

The structure and phase purity of the nanoparticles were confirmed by analysis of the X-ray diffraction (XRD; PANalytical, X’Pert Pro MPD) patterns and the results are presented in Figure 1d. The diffraction peaks matched well with the characteristic peaks of the cubic spinel-type lattice of CoFe_2_O_4_, which in turn is well matched to the standard XRD pattern (JCPDS Card No. 22-1086). The peaks observed at 30.1°, 35.5°, 43.1°, 53.6°, 57.1°, 62.7°, and 74.2° can be assigned to the (220), (311), (400), (422), (511), (440), and (533) planes of spinel CoFe_2_O_4_, respectively. This result indicates that the obtained high-purity CoFe_2_O_4_ nanoparticles have good crystallinity. The average crystallite size of the CoFe_2_O_4_ nanograin was estimated to be approximately 9.25 nm via X-ray line broadening using Scherrer’s equation.

**Figure 2:**
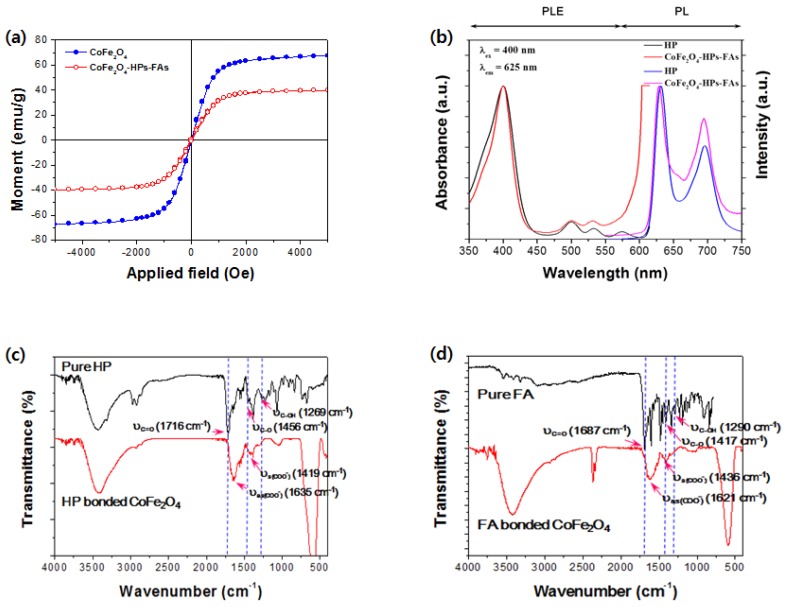
Photophysical and magnetic properties of multifunctional magnetic nanoparticles. (**a**) Room-temperature magnetic hysteresis loops of the CoFe_2_O_4_ nanoparticles and the CoFe_2_O_4_-HPs-FAs; (**b**) photoluminescence and photoluminescence excitation spectra of pure HP and CoFe_2_O_4_-HPs-FAs in THF; FT-IR spectra of (**c**) pure HP and HP bound with CoFe_2_O_4_ and of (**d**) pure FA and FA bonded with CoFe_2_O_4_.

The CoFe_2_O_4_ nanoparticles and CoFe_2_O_4_-HPs-FAs showed good magnetic properties. Figure 2a presents the room-temperature hysteresis loop as a function of the applied magnetic field, or the M versus H curve. The magnetization curves of both samples exhibited no hysteresis, and no coercivity was reached, even at the highest magnetic field applied. This indicates that both magnetic particles show superparamagnetic behavior. The CoFe_2_O_4_ nanoparticles showed a high-saturation magnetization value of 67.3 emu/g, whereas the high-saturation value of the surface-modified CoFe_2_O_4_-HPs-FAs was lower at 39.7 emu/g. The difference in the saturation values is attributed to the diamagnetic contribution of the diamagnetic organic molecules that are chemically bonded to the nanoparticle surface.

From the photoluminescence and photoluminescence excitation spectra shown in Figure 2b, the HP solution showed excitation peaks at 401 (Soret band), 500, 532, and 574 nm (Q band), and the CoFe_2_O_4_-HPs-FAs solution showed the same characteristic peaks. No significant shift in the excitation wavelength was observed in comparison to the dissolved CoFe_2_O_4_-HPs-FAs, suggesting that the HP molecules, as a PS, remained stable after conjugation to the nanoparticles. At the excitation wavelength of 400 nm, the pure HP produced two strong emission peaks located at 631 nm and 696 nm, respectively, and the CoFe_2_O_4_-HPs-FAs exhibited slightly blue-shifted peaks at 628 nm and 694 nm. The blue-shifted emission peaks are attributed to the strong bonding between HP and the magnetic CoFe_2_O_4_ nanoparticles.

To confirm the formation of the metal-organic complex, the Fourier Transform InfraRed (FT-IR) spectra of pure HP, HP-coated CoFe_2_O_4_, and FA-coated CoFe_2_O_4_ nanoparticles were compared. As shown in Figure 2c,d, the absorption peaks were mainly detected in the fingerprint region. Before complex formation, the IR spectra of pure HP and pure FA exhibited a peak in the range of 1687–1716 cm^−1^, indicating the presence of a C=O stretching band of the -COOH groups. In addition, coupled vibrations involving C-O stretching and the O-H deformation (υ_C-OH_) were observed in the range of 1417–1456 cm^−1^ and 1269–1290 cm^−1^, respectively. These results indicate that the pure HP and FA molecules have protonated carboxyl groups (-COOH), as previously described [22]. After the carboxyl acid was converted to the complexes, the IR spectra of HP-coated CoFe_2_O_4_ and FA-coated CoFe_2_O_4_ nanoparticles showed that the absorption bands of the protonated carboxyl groups significantly changed. Three absorption bands corresponding to the stretching vibrations of the C=O group, (C-O), and υ_C-OH_ of the -COOH group at 1260–1720 cm^−1^ disappeared, whereas the bands assigned to asymmetric vibrations υ_as_(COO), at 1621–1635 cm^−1^, and symmetric vibrations υ_s_(COO), at 1419–1436 cm^−1^, appeared. These spectral changes can also be caused by the formation of cation–carboxylate complexes owing to covalent chemical bonding, as described previously [22,23].

The loading capacity with HP molecules of the multifunctional CoFe_2_O_4_-HPs-FAs was determined by UV–Vis spectroscopy (Ultraviolet–visible spectroscopy). From the calculated results, when the CoFe_2_O_4_ nanoparticle weights varied at 1.56, 3.13, 6.25, 12.5, and 25 μg, the weights of the HP molecules bonded to the surfaces of the CoFe_2_O_4_ nanoparticles were 0.2, 0.4, 0.8, 1.60, and 3.22 μg, respectively. Similarly, the concentrations of the FA molecules bonded to the surfaces of the CoFe_2_O_4_ nanoparticles were 0.09, 0.17, 0.35, 0.69, and 1.38 μg according to the weights of the CoFe_2_O_4_ nanoparticles of 1.56, 3.13, 6.25, 12.5, and 25 μg, respectively.

### 2.2. Singlet Oxygen Generation

**Figure 3:**
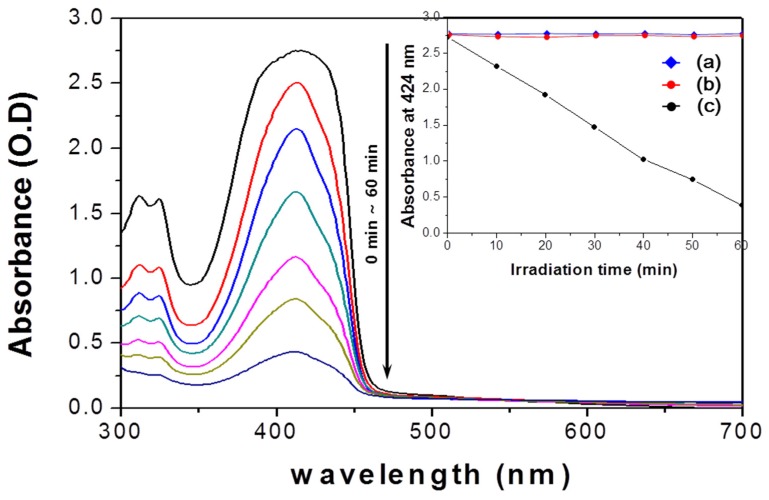
UV–Vis spectra of DPBF according to irradiation time in THF solution with the CoFe_2_O_4_-HPs-FAs under a Xe lamp. The inset presents the absorption (OD) of DPBF in THF at 424 nm as a function of irradiation time. (**a**) DPBF only plus light; (**b**) DPBF with the CoFe_2_O_4_-HPs-FAs without light; (**c**) DPBF with the CoFe_2_O_4_-HPs-FAs plus light.

In a PDT process, absorption of light by PSs eventually results in the generation of singlet oxygen and other ROS. Singlet oxygen is the major cytotoxic species leading to cell death through the so-called type II mechanism [24,25]. To evaluate the capability of ^1^O_2_ generation of CoFe_2_O_4_-HPs-FAs, 1,3-diphenylisobenzofuran (DPBF) was employed as a probe molecule. Figure 3 shows the extensive bleaching of DPBF as a function of time (amplitude reduction of spectral features at 424 nm) when incubated with CoFe_2_O_4_-HPs-FAs in THF and irradiated with a Xe lamp. Control experiments with only DPBF using the same excitation wavelength showed no bleaching. Therefore, the multifunctional magnetic nanoparticles could be a very important PDT reagent.

### 2.3. Biocompatibility of Multifunctional CoFe_2_O_4_-HPs-Fas

**Figure 4:**
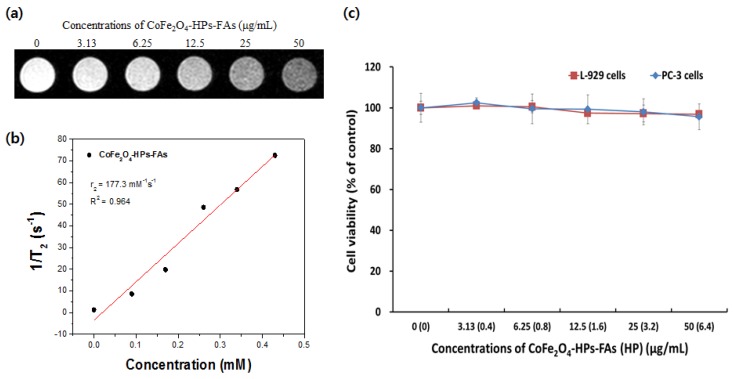
T_2_-weighted MR imaging and biocompatibility of CoFe_2_O_4_-HPs-FAs. (**a**) T_2_-weighted MR images of prostate cancer cells (PC-3 cells) treated with CoFe_2_O_4_-HPs-Fas; (**b**) Plot of T_2_ relaxation rate r_2_ (1/T_2_) for CoFe_2_O_4_-HPs-Fas; (**c**) Cytotoxicity of CoFe_2_O_4_-HPs-FAs (60 nm) in fibroblasts (L-929 cells) and prostate cancer cells (PC-3 cells). Data are expressed as the mean ± standard deviation (*n* = 6).

As superparamagnetic CoFe_2_O_4_ nanoparticles are good T_2_-type (negative) contrast agents in MRI, and FA and HP are biocompatible cancer-targeting and therapeutic agents, the anti-cancer effect of CoFe_2_O_4_-HPs-FAs was investigated by evaluating the MR signal-enhancing property. With increasing concentrations of CoFe_2_O_4_-HPs-FAs in the cells, the MR signal was significantly enhanced (negative in brightness in the T_2_-weighted image) in vitro (Figure 4a). These results indicate that the nanoparticles can generate high magnetic-field gradients near the surface of the CoFe_2_O_4_-HPs-FAs. Additionally, the relaxivity r_2_ (1/T_2_) increases linearly under these conditions (Figure 4b), indicating that the CoFe_2_O_4_-HPs-FAs generated MRI contrasts on T_2_-weighted spin-echo sequences. Transverse relaxivity r_2_ values were determined from the slope of the linear fit to the data points in 1/T_2_ vs. the CoFe_2_O_4_-HPs-FAs concentration plot. The r_2_ value obtained for CoFe_2_O_4_-HPs-FAs was 177.3 mM^−1^s^−1^. As shown in Figure 4a,b, the T_2_-weighted phantom images of the CoFe_2_O_4_-HPs-FAs exhibited a significant negative dose-dependent contrast enhancement, suggesting that these nanoparticles are promising for theragnostic purposes.

To evaluate the biocompatibility of the CoFe_2_O_4_-HPs-FAs, cytotoxicity tests were carried out with fibroblasts (L-929 cell) and prostate cancer cells (PC-3 cells) using a method recommended by the International Organization for Standardization (ISO 10993-5) [26]. As shown in Figure 4b, the viability of both cell types was not decreased when incubated with CoFe_2_O_4_-HPs-FAs as compared to the untreated control cells, and cell viabilities at each concentration of CoFe_2_O_4_-HPs-FAs were more than 95%, indicating that the CoFe_2_O_4_-HPs-FAs have no cytotoxicity in L-929 and PC-3 cells. Collectively, these results demonstrate that CoFe_2_O_4_-HPs-FAs have good biocompatibility and can be used for clinical cancer therapy.

### 2.4. Optimization of the Cellular Uptake and Light Irradiation Time of CoFe_2_O_4_-HPs-Fas

**Figure 5:**
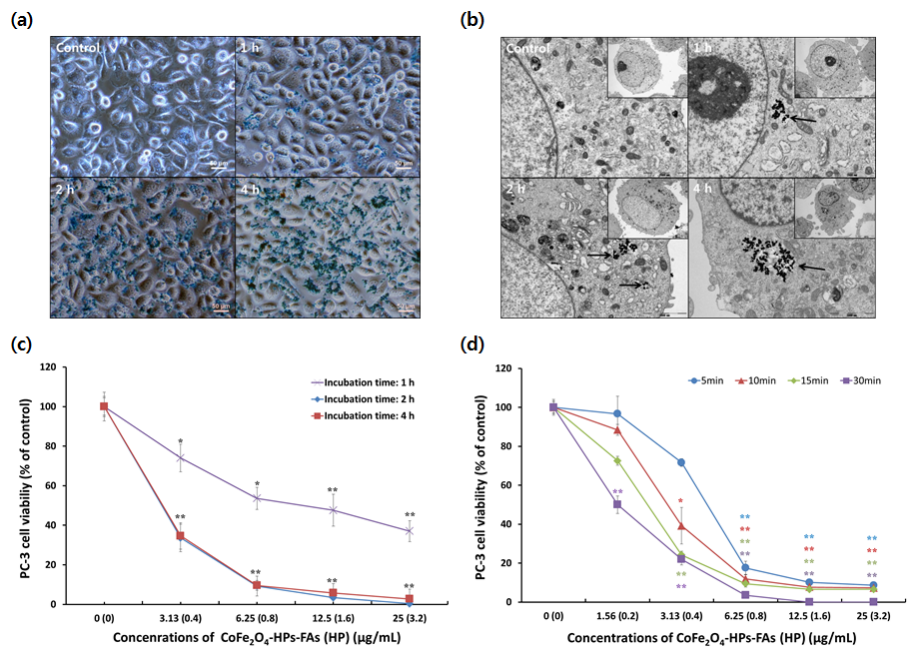
Cellular uptake, intracellular localization, and photodynamic anticancer activities of CoFe_2_O_4_-HPs-FAs in prostate cancer cells (PC-3 cells). (**a**) Microscopic and (**b**) transmission electron microscopic images of CoFe_2_O_4_-HPs-FAs in PC-3 cells to evaluate their cellular uptake and intracellular localization. PC-3 cells treated with 6.25 (0.8) μg/mL CoFe_2_O_4_-HPs-FAs (HPs) were incubated for 1, 2, and 4 h in the dark. The TEM images are magnified from a whole cell image (inset). Black arrows indicate the CoFe_2_O_4_-HPs-FAs. The scale bars represent 50 μm and 2 μm; (**c**) Photodynamic anticancer activity of CoFe_2_O_4_-HPs-FAs according to the incubation time of CoFe_2_O_4_-HPs-FAs with prostate cancer cells (PC-3 cells); (**d**) Photodynamic anticancer activity of CoFe_2_O_4_-HPs-FAs according to the exposure dose of light emitting diode (LED) light to PC-3 cells. Data are expressed as the mean ± standard deviation (*n* = 6) and were analyzed by Student’s *t*-tests. Statistical significance was defined as *p* < 0.05 (* *p* < 0.05, ** *p* < 0.005 vs. control at the same time).

Cellular uptake and the intracellular distribution of the CoFe_2_O_4_-HPs-FAs are the most important factors for their anticancer efficacy by PDT. Therefore, we carried out cell staining with the Prussian blue staining method and TEM analysis after incubating PC-3 prostate cancer cells with the CoFe_2_O_4_-HPs-FAs for 1, 2, and 4 h to confirm the optimal cellular uptake time and intracellular distribution. As shown in Figure 5a, incubation time had a substantial effect on the cellular uptake of the CoFe_2_O_4_-HPs-FAs. The number of CoFe_2_O_4_-HPs-FAs in the cells was proportional to the incubation time and the accumulated CoFe_2_O_4_-HPs-FAs in PC-3 cells appeared to be located in the cytosol. As shown in Figure 5b, the TEM images also clearly demonstrated that most of the CoFe_2_O_4_-HPs-FAs were located in the cytoplasm, and the number of CoFe_2_O_4_-HPs-FAs in the cytoplasm was also increased depending on the incubation time with cells.

To further evaluate the optimal cellular uptake time of the CoFe_2_O_4_-HPs-FAs in prostate cancer cells, the PC-3 cells were incubated with the CoFe_2_O_4_-HPs-FAs for 1, 2, and 4 h, and each cell was irradiated with LED light at a dose of 18.36 J/cm^2^ to confirm the anticancer activity of the CoFe_2_O_4_-HPs-FAs depending on the incubation time. As shown in Figure 5c, the cell viabilities of PC-3 cells were decreased in a dose-dependent manner, regardless of the incubation time of the CoFe_2_O_4_-HPs-FAs with PC-3 cells. The cell viability with 1 h incubation was 100, 74, 53.6, 47.6, and 37.1% with increasing CoFe_2_O_4_-HPs-FA concentrations, respectively. However, the number of viable cells significantly decreased at 2 and 4 h incubation with increasing doses of the CoFe_2_O_4_-HPs-FAs, from 100, 33.6, 9.3, 3.4, and 0.4% for 2 h and from 100, 34.6, 9.6, 8.9, and 5.8% for 4 h compared to control levels. These results suggested that an increased incubation time—i.e., 2 and 4 h—resulted in significantly better photo-killing efficacy of CoFe_2_O_4_-HPs-FAs in PC-3 cells compared with a 1-h incubation time. Moreover, the photodynamic anticancer activity at 2 h of incubation was higher than that at 4 h of incubation at high concentrations (12.5 (1.60) and 25 (3.22) μg/mL) of CoFe_2_O_4_-HPs-FAs (HPs). Specifically, the photo-killing efficacy of 12.5 (1.60) and 25 (3.22) μg/mL CoFe_2_O_4_-HPs-FAs (HPs) ranged from over 96% (*p* < 0.005) to almost 100%. These results confirmed a close correlation between cellular uptake time and anticancer efficacy by PDT, although there was no difference in the photo-killing efficacy between 2 h and 4 h of incubation. Therefore, we selected 2 h as the optimal incubation time for the subsequent photodynamic anticancer activity test of the CoFe_2_O_4_-HPs-FAs.

### 2.5. Anticancer Activity of CoFe_2_O_4_-HPs-Fas

To confirm the photodynamic anticancer activity according to the exposure dose of LED light exposed to PC-3 cells, the cells were incubated with various concentrations of the CoFe_2_O_4_-HPs-FAs and irradiated by LED light at doses of 3.06, 6.12, 9.18, and 18.36 J/cm^2^ after incubation for 2 h. The photo-killing efficacy was also quantified using the Cell Counting Kit-8 (CCK-8) method, as shown in Figure 5d. The photodynamic anticancer activities by LED irradiation were significantly increased under each dose, even at the lowest dose of 3.06 J/cm^2^, and cell viabilities dramatically decreased with increased concentrations of the CoFe_2_O_4_-HPs-FAs. The photo-killing efficacies of the CoFe_2_O_4_-HPs-FAs were markedly increased in a dose-dependent manner. These results demonstrated a close correlation between exposure dose of light and dose of the CoFe_2_O_4_-HPs-FAs on the photo-killing efficacy.

Finally, to confirm the anticancer mechanism by PDT in PC-3 cells, we conducted morphological analysis to evaluate the rate of apoptotic cell death induced by LED irradiation after incubation with the CoFe_2_O_4_-HPs-FAs for 2 h using an Annexin V-fluorescein isothiocyanate (FITC) apoptosis detection kit and Hoechst 33342 fluorescence dye. Annexin V is an intracellular protein that binds to phosphatidylserine. Phosphatidylserine is normally only found on the intracellular face of the plasma membrane in healthy cells, whereas during the early stage of apoptosis, membrane asymmetry is lost and phosphatidylserine translocates to an external site. Therefore, FITC-labeled Annexin V can be used to specifically target and identify apoptotic cells.

**Figure 6:**
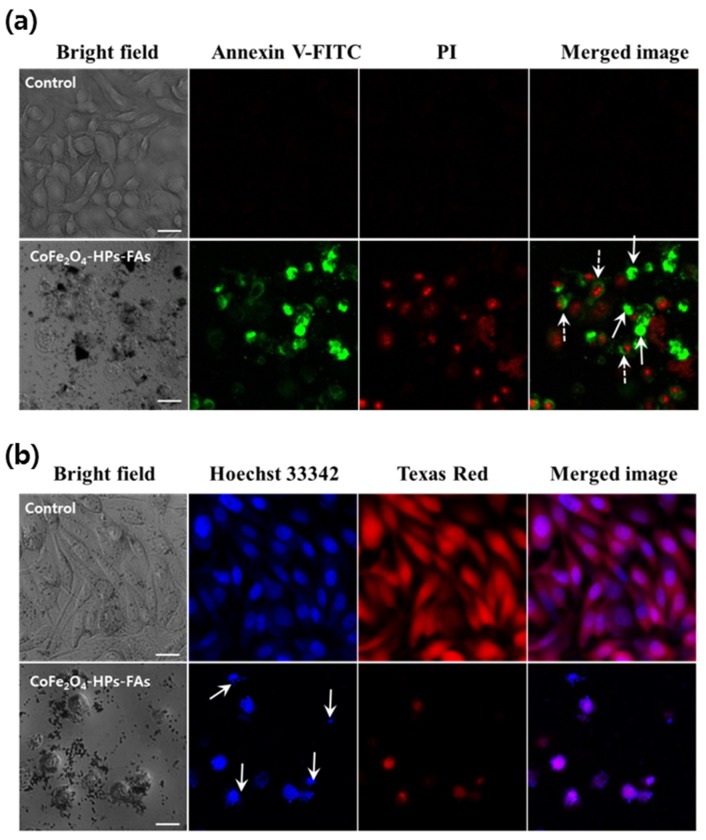
Confocal fluorescence images for cell membrane translocation and nuclear fragmentation in apoptotic PC-3 cells. (**a**) Confocal fluorescence images for cell membrane translocation stained with Annexin V-FITC (green) for the cell membrane and propidium iodide (PI, red) for the nucleus in PC-3 cells at 6 h post-irradiation. Cells stained green are apoptotic cells, and solid line and dashed arrows indicate early- and late-stage apoptotic cells, respectively; (**b**) Confocal fluorescence images for nuclear fragmentation (solid line arrows) stained with Hoechst 33342 (blue) for the nucleus and with Texas Red (red) for whole cells in PC-3 cells at 6 h post-irradiation. Apoptotic cell death was induced by LED irradiation at an irradiation dose of 18.36 J/cm^2^ after treatment with 6.25 (0.8) μg/mL CoFe_2_O_4_-HPs-FAs (HPs) for 2 h. The scale bar represents 25 μm.

Figure 6a shows the stained images of normal and apoptotic cells at 2 h post-irradiation. In the control (untreated), cells stained by Annexin V-FITC and propidium iodide (PI) were not detected, whereas the cells treated with the CoFe_2_O_4_-HPs-FAs were stained green by Annexin V-FITC and red by PI. Cells showing only green fluorescence indicated early-stage apoptotic cells for cell membrane translocation, whereas double-stained cells in green and red indicated late-stage apoptotic cells. The results demonstrated that LED irradiation after treatment of the CoFe_2_O_4_-HPs-FAs to cancer cells is mediated by the induction of cell death, and the majority of cell death was mediated via apoptosis.

Moreover, we evaluated the extent of nucleus fragmentation in PC-3 cells with Hoechst 33342 dye. As shown in Figure 6b, the cells irradiated with LED light after treatment of the CoFe_2_O_4_-HPs-FAs for 2 h were quickly condensed and some showed a granular nucleus body, which was not detected in the control cells.

These results are consistent with the results of photodynamic anticancer efficacy shown in Figure 5c,d, further indicating that the photodynamic anticancer effects may be induced via apoptosis.

## 3. Materials and Methods

### 3.1. Synthesis of Multifunctional CoFe_2_O_4_-HPs-Fas

The CoFe_2_O_4_ magnetic nanoparticles were prepared by applying a similar method as that described in the previous report [27]. In brief, 0.36 g FeCl_3_·6H_2_O, 0.11 g CoCl_2_·2H_2_O, and 1.5 g NaOAc were dissolved in ethylene glycol/diethylene glycol mixture solvent (5:15, *v*/*v*), and then this mixture was vigorously stirred for 30 min. The solution was transferred to an 80-mL Teflon-lined autoclave, which was sealed and maintained at 200 °C for 10 h, and was then cooled to room temperature naturally. The black precipitation was collected by magnetic decantation, washed with deionized water and absolute alcohol several times, and then dried in a vacuum oven at 60 °C for 12 h.

To conjugate more PS molecules, the surfaces of the CoFe_2_O_4_ nanoparticles were treated with micro-dielectric barrier discharge plasma for 30 min according to a previously reported method [22].

The photo-functionality and targeting functionality on the CoFe_2_O_4_ nanoparticles were achieved using a wet chemical process similar to the method described in our previous report [28].

### 3.2. Characterization of Multifunctional CoFe_2_O_4_-HPs-Fas

Field-emission scanning electron microscopy (FE-SEM; SU-70, Hitachi, Tokyo, Japan) and transmission electron microscopy (TEM; JEM-2100F, JEOL, Tokyo, Japan) were applied to determine the size and surface morphology of the multifunctional sub-micron particles. The X-ray diffraction (XRD; X’ Pert Pro MPD, PANalytical, Almelo, The Netherlands) pattern of the product was determined on a PANalytical Pert Pro MPD X-ray diffractometer with a Cu Kα radiation source (*λ* = 0.15405 nm) operated at 40 kV and 150 mA in a 2θ range of 20–80°. A vibrating sample magnetometer (VSM; Lakeshore 7300, Lake Shore Cryotronics, Westerville, OH, USA) was utilized to measure the magnetization versus magnetic field loop at room temperature up to 10 kOe. Photoluminescence and photoluminescence excitation spectra were measured on a spectrophotometer (F-4500, Hitachi, Tokyo, JApan). Infrared (IR) spectra were obtained using a Fourier transform (FT)-IR spectrometer (Spectrum 100, Perkin-Elmer, Waltham, MA, USA). For IR measurements, samples were prepared in an agate mortar and then prepared in the form of pressed wafers (ca. 1% sample in KBr).

### 3.3. Detection of Singlet Oxygen

Degradation of 1,3-diphenylisobenzofuran (DPBF) as a singlet oxygen quencher was applied to determine the release of singlet oxygen into the solution [29]. In the photochemical experiment, an aliquot of 3.0075 mL of tetrahydrofuran (THF) solution containing the CoFe_2_O_4_-HPs-FAs and DPBF (4.61 × 10^−8^ M) was introduced to a 1-cm quartz cell in the dark. The light source was a Xe lamp (150 W, Abet Technologies, Milford, MA, USA). A 480-nm glass cutoff filter was used to remove the ultraviolet (UV) light, which prevents direct photodegradation of DPBF. Photodegradation of DPBF was monitored by recording the optical density (OD) of the absorption peak at 424 nm. At every 10 min of irradiation, the absorption spectra of the samples were monitored on a UV–Vis spectrophotometer.

### 3.4. Magnetic Resonance Imaging (MRI) Analysis In Vitro

All MRI experiments were performed on a 3.0-Tesla whole-body MRI scanner (Philips Achieva X-series, Amsterdam, The Netherlands) using a dedicated phased array receiver coil for high-resolution MRI as previously described [22]. In brief, PC-3 cells (a prostate cancer cell line) pre-cultured for 24 h were incubated with various concentrations (0, 3.13, 6.25, 12.5, 25, and 50 μg/mL) of the CoFe_2_O_4_-HPs-FAs for 2 h in a 24-well culture plate. The cells were then fixed with a 2% glutaraldehyde and paraformaldehyde solution and mixed with a 1.5% agar solution in 1.5-mL micro-centrifuge tubes for MRI.

### 3.5. Biocompatibility of Multifunctional CoFe_2_O_4_-HPs-Fas

Cellular toxicity tests on fibroblasts (L-929 cells) and prostate cancer cells (PC-3 cells) were carried out to confirm the biocompatibility of the CoFe_2_O_4_-HPs-FAs, as previously described [22,30,31,32]. In brief, each cell type was plated in a 24-well plate at 2.0 × 10^5^ cells/mL for L-929 cells and at 1.0 × 10^5^ cells/mL for PC-3 cells, incubated at 37 °C in 5% CO_2_ for 24 h, treated with various concentrations (0 (0), 3.13 (0.4), 6.25 (0.8), 12.5 (1.60), 25 (3.22), and 50 (6.44) μg/mL) of CoFe_2_O_4_-HPs-FAs (HPs), and then incubated at 37 °C in 5% CO_2_ for a further 24 h under dark conditions. After incubation, the cells were washed three times with phosphate-buffer saline (PBS) and their viabilities were determined using Cell Counting Kit-8 (CCK-8, Dojindo Laboratories, Kumamoto, Japan). The OD value of each well was measured at 450 nm with a microplate reader (Synergy^TM^ HT, BioTek Instruments, Inc., Winooski, VT, USA) and the cell viabilities are presented as the survival percentage relative to the untreated control.

### 3.6. Optimization of the Cellular Uptake and Light Irradiation Time for Photodynamic Anticancer Activity of Multifunctional CoFe_2_O_4_-HPs-Fas

To confirm the optimal cellular uptake time of CoFe_2_O_4_-HPs-FAs into the cells, PC-3 cells were plated in a 24-well plate at 1.0 × 10^5^ cells/mL and incubated at 37 °C in 5% CO_2_ for 24 h. The cells were further incubated with different concentrations (0 (0), 3.13 (0.4), 6.25 (0.8), 12.5 (1.60), and 25 (3.22) μg/mL) of CoFe_2_O_4_-HPs-FAs (HPs) for 1, 2, and 4 h under dark conditions. The cells were washed three times with PBS, the medium was refreshed, and the cells were irradiated by a general green light-emitting diode (LED) at 18.36 J/cm^2^ as previously reported [22,23,30,31,32]. The LED had a wavelength range of 480–580 nm, a maximum wavelength of 515 nm, and a maximum dose of 18.36 J/cm^2^. After irradiation, the cells were incubated for another 24 h and the following day, their viabilities were measured using the CCK-8 kit as described above.

To confirm the cellular uptake and intracellular localization of CoFe_2_O_4_-HPs-FAs in PC-3 cell with image analysis, two kinds of methods were adopted: Prussian blue staining for cellular uptake and TEM analysis for intracellular localization, as previously described [22]. In brief, PC-3 cells treated with CoFe_2_O_4_-HPs-FAs (6.25 μg/mL) were washed with PBS three times after incubating for 1, 2, and 4 h, and fixed with ice-cold acetone for 10 min to stain the cells with Prussian blue staining reagents. The fixed cells were stained with a 2% potassium ferrocyanide II and 1 M hydrochloric acid mixture (ratio 1:1) for 10 min at 37 °C after washing with PBS, and subsequently counterstained with a 0.1% solution of Nuclear Fast Red in distilled water with 5% aluminum sulfate for 1 min after washing again with PBS. Finally, the stained cells were observed with an inverted microscope (Eclipse Ti, Nikon, Instruments, Inc., New York, NY, USA) after washing with distilled water. To evaluate the intracellular localization of CoFe_2_O_4_-HPs-FAs in prostate cancer cells, PC-3 cells treated with CoFe_2_O_4_-HPs-FAs (6.25 μg/mL) for 1, 2, and 4 h were washed with PBS three times and fixed with 2.5% glutaraldehyde and 4% formaldehyde for 4 h at 4 °C. Fixed cells were washed with PBS twice and treated with 2% osmium tetroxide for 2 h at 4 °C. The fixed cells were washed again with PBS and dehydrated with increasing concentrations of ethanol and embedded in araldite. The fixed PC-3 cells were then observed with TEM (JEM-1011, JEOL, Tokyo, Japan).

To evaluate the appropriate irradiation energy with green LED for targeting the cancer cells, PC-3 cells were plated in a 24-well plate and incubated for 24 h as described above. After incubation, the cells were incubated with different concentrations (0 (0), 1.56 (0.2), 3.13 (0.4), 6.25 (0.8), 12.5 (1.60), and 25 (3.22) μg/mL) of CoFe_2_O_4_-HPs-FAs (HPs) for 2 h and further incubated for 24 h after irradiation at doses of 3.06, 6.12, 9.18, and 18.36 J/cm^2^. The following day, the cell viabilities were measured using the CCK-8 kit as described above.

### 3.7. Morphological Analysis of Apoptotic Cell Death in Prostate Cancer Cells

To confirm the cell death after LED irradiation, the cell membranes and nuclei were stained with an EzWay^TM^ Annexin V-FITC apoptosis detection kit (K29100, Komabiotech Inc., Seoul, Korea) and Texas Red C2-maleimide dye. The cell images were taken by a laser scanning microscope (LSM 700, Carl Zeiss, Oberkochen, Germany) with fluorescence optics (excitation at 488 nm for FITC, 530 nm for PI, 595 nm for Texas Red C2-maleimide, and 352 nm for Hoechst 33342; emission at 518 nm for FITC, 615 nm for Texas Red C2-maleimide, and 620 nm for PI) as previously described [22,23].

### 3.8. Statistical Analysis

All quantitative data (*n* = 6) are expressed as the means ± standard deviation, and statistical comparisons were evaluated with a Student’s *t*-test. Significant differences were indicated by *p* < 0.05.

## 4. Conclusions

In the present study, we synthesized novel FA- and HP-conjugated multifunctional magnetic nanoparticles (CoFe_2_O_4_-HPs-FAs), which were characterized as an effective anticancer reagent for PDT, and demonstrated the dependency of the photodynamic anticancer activities on the incubation time and the exposure dose of LED light in prostate cancer cells (PC-3 cells). These results indicate that the same fluence at different exposure doses results in dissimilar levels of anticancer activities on PC-3 cancer cells as well as in ROS formation. In addition, the increase of the fluence showed an improvement for cell photo-inactivation.
